# Impact of the Morphology of Micro- and Nanosized Powder Mixtures on the Microstructure of Mg-Mg_2_Si-CNT Composite Sinters

**DOI:** 10.3390/ma12193242

**Published:** 2019-10-04

**Authors:** Anita Olszówka-Myalska, Patryk Wrześniowski, Hanna Myalska, Marcin Godzierz, Dariusz Kuc

**Affiliations:** Institute of Materials Science, Department of Material Science and Metallurgy, Silesian University of Technology, Krasińskiego 8, 40-019 Katowice, Poland; patryk.wrzesniowski@polsl.pl (P.W.); hanna.myalska@polsl.pl (H.M.); marcin.godzierz@polsl.pl (M.G.); dariusz.kuc@polsl.pl (D.K.)

**Keywords:** heterophase magnesium matrix composite, Mg_2_Si, carbon nanotubes, nanopowders de-agglomeration, sintering

## Abstract

The problem of preparing a ternary powder mixture, which was meant to fabricate sintered heterophase composite, and consisted of micro- and two nanosized powders, was analyzed. The microsized powder was a pure magnesium, and as nanocomponents, a silicon powder (nSi) and carbon nanotubes (CNTs) with 2% and 1% volume fractions, respectively, were applied. The powder mixtures were prepared using ultrasonic and mechanical mixing in technological fluid, and four mixing variants were applied. The morphology of the powder mixtures was characterized with scanning electron microscopy (SEM), and then, composite sinters were fabricated in a vacuum with hot temperature pressing at 580 °C under 15 MPa pressure, using a Degussa press. The reaction between the nSi and the Mg matrix, which caused the creation of the Mg_2_Si phase in the fabricated Mg-Mg_2_Si-CNT composite, was confirmed with X-ray diffraction (XRD). The porosity and hardness of the composite sinters were examined, and optical microscopy (OM) and quantitative image analyses were carried out to characterize the microstructure of the composites. In the manufacturing process of the Mg-nSi-CNT mixtures, the best results were the following: first separate de-agglomeration of nanocomponents, then their common mixing, and finally, the deposition of nanocomponents at the surface of the microsized magnesium powder. The applied procedure ensured the uniform layer formation of de-agglomerated nanocomponents on the Mg powder, without re-agglomerated nSi and CNTs. Moreover, this type of powder mixture morphology allows to obtain sinters with lower porosity and higher hardness, which is accompanied by precipitation of a finer Mg_2_Si phase. In the Mg-Mg_2_Si-CNT composite, the carbon phase was present, and it was located in the magnesium matrix and in silicide.

## 1. Introduction

Development in the fabrication of nanopowder materials, and their increased availability, is gaining momentum around the world. Nanopowder materials, such as carbon nanotubes (CNTs) [1,2,3,4,5], graphene [5,6,7], and nanosilver [8,9], due to their extraordinary properties, are applied as reinforcing components in composites with polymer [10,11,12], metal [13,14,15,16,17], and ceramic matrices [18,19]. Research focused on the use of nanocomponents concerns composites with the magnesium matrix, due to the low density of this metal [20,21,22]. However, proper use of the unique properties of nanocomponents is limited, due to the components’ high tendency to agglomerate, which means that the consolidation of agglomerated nanofibers or nanoparticles with the matrix component can cause a decrease in, for example, mechanical properties, as well as thermal or electric conductivity. In the case of metal matrix composites manufactured with powder metallurgy methods, powder mixtures with 0.5–4.0 vol.% of nanocomponents are usually applied, and much attention has focused on the formation of homogenous mixtures. Mixing in the ball mills is a method well-known in the literature for preparing powder mixtures, but this method can cause a loss of nanocomponents, the generation of internal defects and their re-agglomeration. Another solution, which has been applied to Mg-CNTs [23,24,25] and Mg-nSiO_2_ [26,27] mixtures, is ultrasonic mixing in technological fluids.

In the present work, ultrasonic mixing was applied to develop a beneficial procedure for preparing ternary mixtures, for a system that included a microsized powder and two nanopowders. That issue is crucial in the design of a new multiphase material technology. In the experiments, carbon nanotubes and silicon nanopowder (nSi) were used. During sintering, the silicon reacts with magnesium, and an intermetallic Mg_2_Si phase is formed [28,29,30,31,32]. Thus, from the Mg-nSi-CNT powder mixture, a composite with Mg_2_Si particles and nanotubes will be created. Due to the mechanical properties, magnesium silicide is often used as a reinforcement phase in ex situ and in situ composites with aluminum [33,34,35,36] and the magnesium [26,27,28,29,30] matrix. The intermetallic phase also exhibits good thermoelectric properties, and many studies have focused on that aspect of application [29,30]. However, the structural effect of the formation of Mg_2_Si in an environment enriched with CNTs is not known in the literature. Some movement of CNTs induced by synthesis reaction can be expected in the composite. The thermodynamic data indicate the reaction 2Mg + Si→ Mg_2_Si, and Gibbs free energy ΔG at 580 °C (the calculations were carried out using HSC Chemistry 4) reaches the value of −71.76 kJ. However, transformation of the CNTs into SiC is also possible, because ΔG for the reaction Si + C→SiC is also negative, and equals −64.96 kJ.

In the here presented studies, during the preparation of the ternary powder mixtures, it was assumed that de-agglomeration of the main nanocomponents would occur due to the ultrasound action in technological liquid (alcohol) [37] supported by mechanical mixing. The issue that requires detailed experiments and analyses is the correct order of the mixing steps which may limit uncontrolled re-agglomeration of the nanocomponents. Four variants of the mixing procedure were applied, and the effectiveness of the de-agglomeration was evaluated based on the powder mixture morphology. The porosity, hardness and microstructure of the composite sinters were also examined. Special attention was paid to Mg_2_Si phase dispersion, because the presence of nSi clusters in a ternary powder mixture induces relatively massive silicides in the composite, and on this basis, the effectiveness of the de-agglomeration of the nanocomponents can be characterized. The work concerns the general problem as the simultaneous use of fibrous and particulate nanocomponents in the conventional powder metallurgy processes, where a microsized powder is the main ingredient. This issue will occur in materials design regardless of the components chemical and phase composition. The powder ultrasonic mixing used in technological procedure allows to de-agglomerate primary nanofibers and nanoparticles agglomerates, and then their mixture with microsized component formation. The morphology of the mixture is controlled by interaction processes occurring in systems: nanofiber-microparticle, nanoparticle-microparticle, and nanofiber-nanoparticle. This type of powder mixture can be potentially applied to fabricate sintered composites or semi-products, in form of cold-pressed or hot-pressed moulds. In the literature, an application of multiphase moulds obtained by powder metallurgy was proposed for metal matrix composite fabrication with the different methods of plastic working processes [38,39,40,41,42,43,44] or casting methods [45,46,47]. The goal of such technological solutions is an increase of the reinforcing phases homogeneity, and its dispersion, in the case of in situ formed phases.

## 2. Materials and Methods 

**Figure 1:**
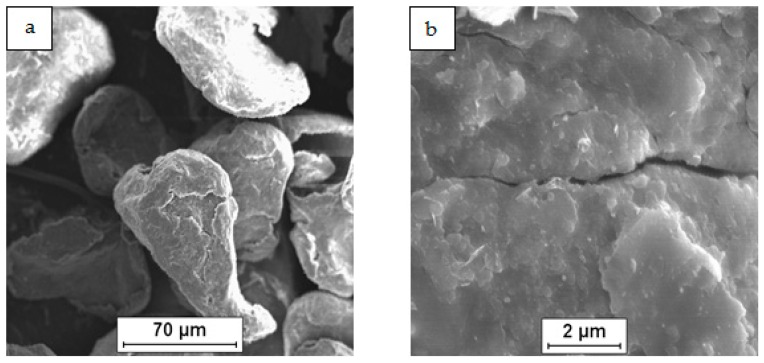
SEM micrographs of Mg powder as-fabricated: (**a**) particles, (**b**) surface of single particle.

**Figure 2:**
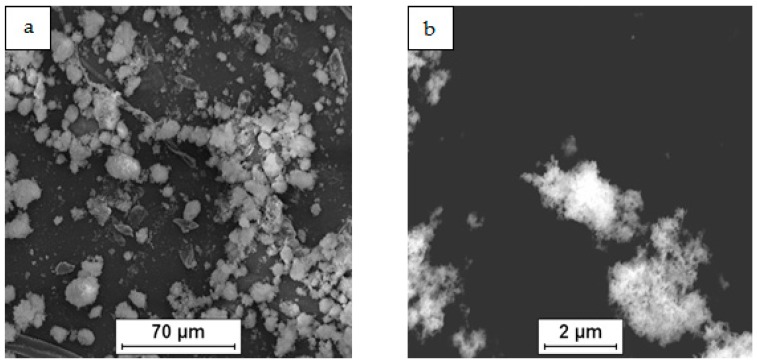
SEM micrographs of silicon nanopowder (nSi) as-fabricated: (**a**) agglomerates of different size, (**b**) morphology of single agglomerate.

**Figure 3:**
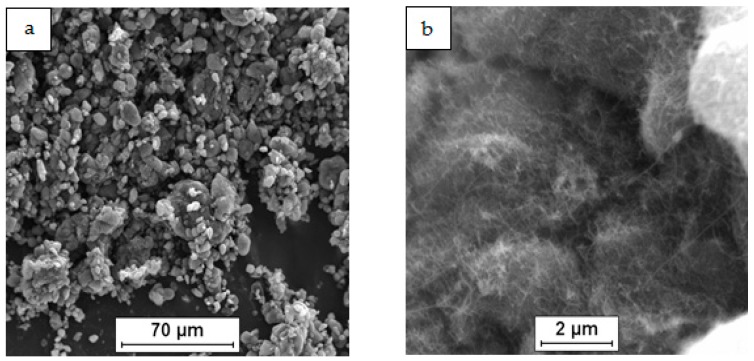
SEM micrographs of as-fabricated carbon nanotubes (CNT): (**a**) agglomerates of different size, (**b**) morphology of single agglomerate.

As a raw material in the experiments, magnesium powder (63–250 µm, Sigma Aldrich, 13112, Saint Louis, MO, USA), silicon nanopowder (average particle size 50 nm, Sigma Aldrich, Saint Louis, MO, USA) and carbon nanotubes (diameter 50–85 nm, length 10–15 µm, Multi-Walled Carbon Nanotube Powder, GRAPHENE SUPERMARKET, Ronkonkoma, State NY, US) were applied. Micrographs of the fabricated Mg, nSi and CNT powders observed with a scanning electron microscope (SEM) are presented in Figure 1, Figure 2 and Figure 3. The surface of the magnesium granules (Figure 1) is irregular, which might be a beneficial feature during the mixing process and the deposition of the nanocomposites on a single granule. The SEM micrographs of nSi (Figure 2) and the CNTs (Figure 3) (raw materials) revealed a few or dozen micrometer-sized clusters, and that disadvantage indicates the necessity of de-agglomeration in processing ternary powder mixtures. Otherwise, correct exploitation of unique nanoaddition properties will be ineffective, due to the submicro- and nanopores between single particles or fibers consolidated with the metal matrix, independent of the technology.

In the experiments, the same composition of the powder mixture, 97% Mg; 2% nSi; 1% CNT (vol.%), was chosen. Based on previous experiences regarding preparation of mixtures with nanocomponents [26,27,33], four variants of mixture processing consisting of ultrasonic and mechanical mixing in alcohol were applied (Table 1). The main differences were in the sequence of de-agglomeration of the nanocomponents and then mixing with the microsized powder. When the mixing procedure was completed, the liquid alcohol was removed from the suspension, and then the powder mixture was dried for 18 h at 60 °C.

The microstructure of the ternary powder mixtures was examined with a field emission (FE)SEM (FE Hitachi S-4200, Hitachi Group, Tokyo, Japan), and the distribution of the components was analyzed. Then the Mg-nSi-CNT powder mixtures and pure Mg powder as a reference material were sintered in a Degussa press, in a vacuum atmosphere, at 580 °C under 15 MPa pressure. Sinters 20 mm in diameter and 10 mm high were obtained.

The porosity measurements of the fabricated composite samples were carried out with the Archimedes method, and Vickers hardness HV0.2 was determined with the Zwick 110 hardness tester (Zwick, Ulm, Germany). For the phase composition examination of the sinters, X-ray diffraction (XRD) was applied (X’Pert 3 Powder X- ray diffractometer, Malvern Panalytical Ltd., Royston, UK). For microstructure characterization, polished samples without additional etching were prepared. The observations were carried out with an optical microscope (OM; GX71 Olympus, Tokyo, Japan) and scanning electron microscope (SEM, Hitachi 3400N, Tokyo, Japan) equipped with wavelength-dispersive X-ray spectroscope (WDS Thermo Scientific Magna Ray, Waltham, Massachusetts, US). By WDS method, the elemental mapping of magnesium (TAP crystal), silicon (TAP crystal), carbon (NiC80 crystal), and oxygen (NiC80 crystal) were obtained. The quantitative metallography examinations were focused on Mg_2_Si phase characterization, the volume fraction evaluated with the area fraction and dispersion evaluated with the particle cross-section area. The examinations were performed using Met-Ilo software (J. Szala, Silesian University of Technology, Katowice, Poland), to analyze the influence of the morphology of the Mg-nSi-CNT powder mixture on the microstructure of the composite sinter. The stereological parameters were determined using OM images, although the resolution of method excludes identification of a single CNT, and allows observation only of bigger objects, due to the characteristic blue of the Mg_2_Si phase, identifying this silicide was very simple, in contrast to the SEM observation. The size of the Mg_2_Si phase particles and the quantity were measured, and then the particles divided into five size classes (left closed intervals: 10–100, 100–1000, 1000–10,000, 10,000–100,000 and > 100,000 µm^2^). The measurement procedure was conducted in a relatively large area (15 different areas were chosen for investigation, and they were conducted at 50X magnification). The image analyses allowed to indicate the most effective mixing method for preventing the re-agglomeration phenomenon. In the case of this phenomenon, the methodology is more accurate in comparison with thin foils, for example, where only a small area of the material can be analyzed. Additionally, the silicide particles detected with the OM, and characterized with quantitative metallography, were only a portion of the nSi and Mg reaction product. The other particles were smaller, even nanosized, and more difficult to distinguish.

## 3. Results

### 3.1. Characterization of the Powder Mixtures

Examinations of the Mg-nSi-CNT powder mixtures with SEM revealed that the main effect of the mixing procedure was the deposition of the nanopowders on the Mg powder. This phenomenon is known from the literature for a binary mixture of microsized and nanosized components and occurs due to adhesion forces [23,24,25,26,27,28]. Moreover, the macroscopic observations of the mixtures did not reveal a significant residue of nanocomponents on the mixer walls after mixing, and generally, the metallic color of the magnesium granules disappeared. Additionally, the technological liquid was transparent after the process.

**Figure 4:**
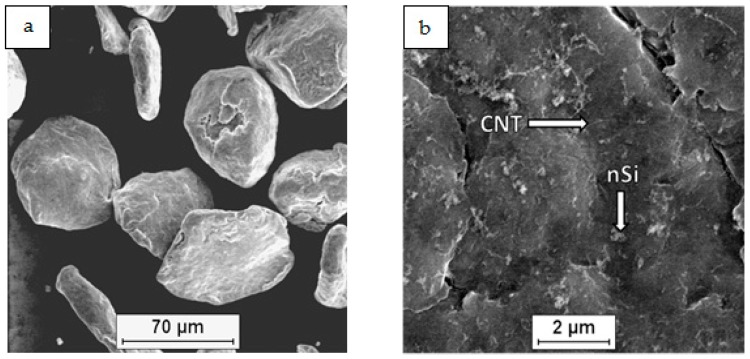
SEM micrographs of Mg+(nSi_D_+CNT_D_) powder mixture: (**a**) morphology of powder mixture, (**b**) surface of microsized Mg coated with CNT and nSi.

**Figure 5:**
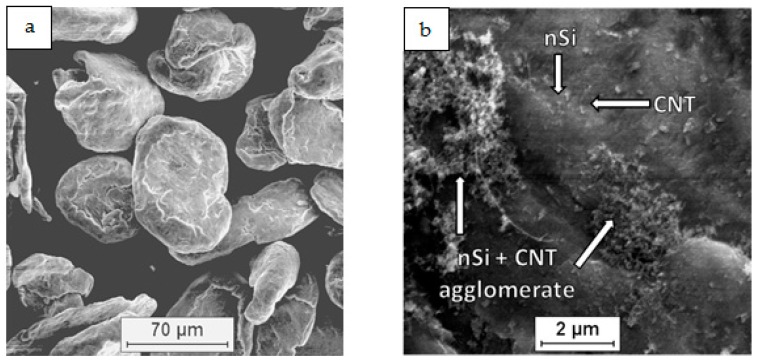
SEM micrographs of Mg + (nSi + CNT)_D_ powder mixture: (**a**) morphology of powder mixture, (**b**) surface of microsized Mg coated with CNT and nSi.

**Figure 6:**
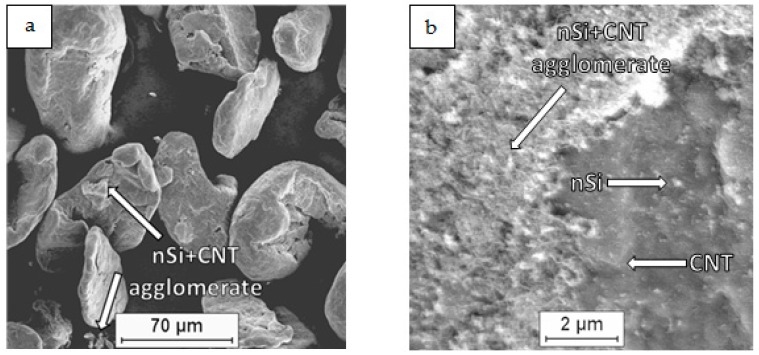
SEM micrographs of (Mg + CNT_D_) + nSi_D_ powder mixture: (**a**) morphology of powder mixture, (**b**) surface of microsized Mg coated with CNT and nSi.

**Figure 7:**
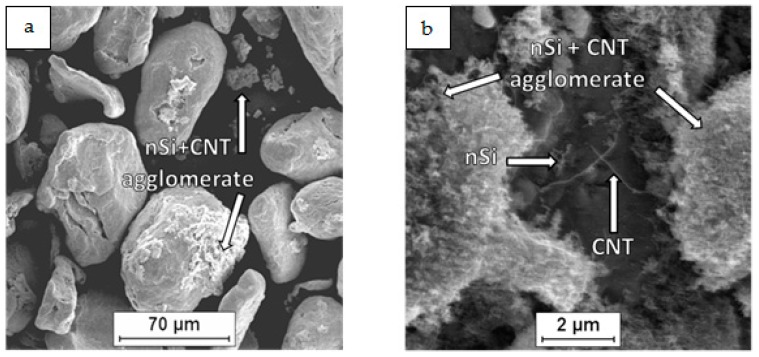
SEM micrographs of (Mg + nS_D_) + CNT_D_ powder mixture: (**a**) morphology of powder mixture, (**b**) surface of microsized Mg coated with CNT and nSi.

The microscopic examination of the dried ternary powder mixtures (Figure 4, Figure 5, Figure 6 and Figure 7) showed differences in the distribution of the CNTs and nSi, which was related to the mixing procedure. Magnesium powder was coated with nanocomponents, but an unfavorable phenomenon was observed in the case of the (Mg + nSi_D_) + CNT_D_ and (Mg + CNT_D_) + nSi_D_ samples (Figure 6a and Figure 7a). Agglomerates of CNTs and nSi, a few micrometers and larger, were detected, and they were poorly connected to the Mg grains, and even separate from the metallic granules. This effect was more intense for the (Mg + nSi)_D_ + CNT_D_ mixture, but in general, the tendency to re-agglomeration of two different nanocomponents in the ultrasonic mixed suspension was noticed. It occurred when the microsized component (Mg) was not mixed at the same time with the two previously de-agglomerated nanocomponents. The main reason for that type of re-agglomeration likely is the fibrous morphology of the CNTs. In the procedure for mixing the (Mg + nSi_D_) + CNT_D_ powder, the nSi particles initially deposited at magnesium were caught by the de-agglomerated fibers, while in the processes for preparing (Mg + CNT_D_) + nSi_D_, the nSi accumulated around the CNTs.

Moreover, the microstructure observations indicated that in the proposed mixing procedures, for one micro- and two nanosized components, selective deposition of previously de-agglomerated nanocomponents could not be expected. This means that the formation of a layered structure, where first, nSi covers the Mg particles, and then the CNTs create another layer, or vice versa, cannot be achieved. That excludes formation of the CNT nanozone at the Mg-nSi interface, and limits further de-agglomeration of the fibrous component, induced in conditions where the silicide forms not by reactive diffusion, but by self-propagating high-temperature synthesis (SHS).

In comparison with the powder mixtures described previously, the results of the FE-SEM examinations for the two other mixtures, Mg + (nSi + CNT)_D_ and Mg + (nSi_D_ + CNT_D_), revealed some differences in the microstructure. Generally, the Mg surface was coated with distinctly separate CNT and nSi grains, and the layer of nanocomponents was uniform and relatively thin (Figure 4b). However, when the CNT and nSi were de-agglomerated together before being introduced in the Mg-alcohol suspension (the Mg + (nSi + CNT)_D_ mixture), the agglomerates of CNT + nSi at the Mg surface were revealed. That effect may confirm a tendency of CNTs and nSi to mutual agglomeration, induced by the fibrous component.

### 3.2. Characterization of Composite Sinters

**Figure 8:**
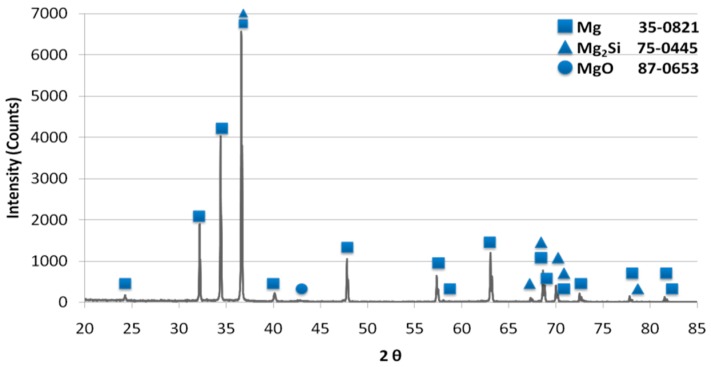
X-Ray Diffraction Pattern of Diffraction Mg + nSi_D_ + CNT_D_ composite.

An example of the XRD pattern obtained for the composites is shown in Figure 8, and it confirms the presence of αMg and a new Mg_2_Si phase, formed in situ as a result of the nSi reaction with the Mg matrix. A weak signal coming from MgO phase was identified as well. The presence of a carbon component, or SiC, was not detected due to the low carbon component content and insufficient method sensitivity. Therefore, in a future investigation, the identification of nanostructural phases in composite will be performed, and the high-resolution transmission electron microscopy and selective area electron diffraction methods will be conducted.

**Figure 9:**
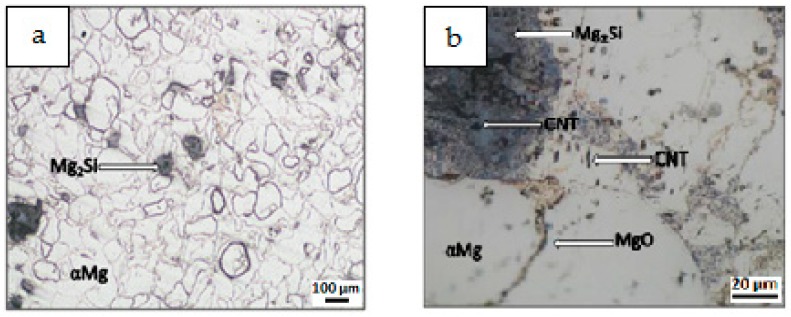
OM micrographs of Mg-Mg_2_Si-CNT composite fabricated from Mg+(nSi_D_+CNT_D_) powder mixture: (**a**) Mg_2_Si agglomerates in Mg matrix, (**b**) phases of different size and morphology.

**Figure 10:**
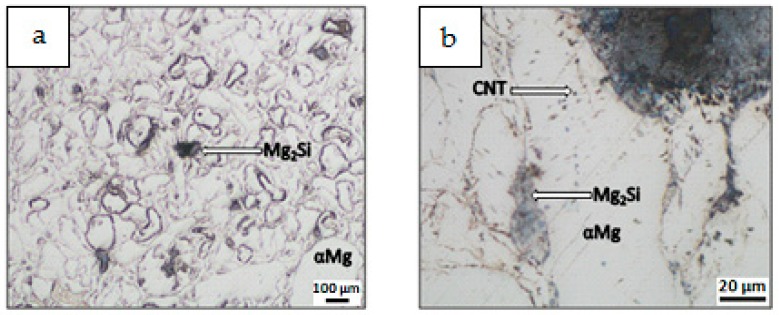
OM micrographs of Mg-Mg_2_Si-CNT composite fabricated from Mg + (nSi + CNT)_D_ powder mixture: (**a**) Mg_2_Si agglomerates in Mg matrix, (**b**) phases of different size and morphology.

**Figure 11:**
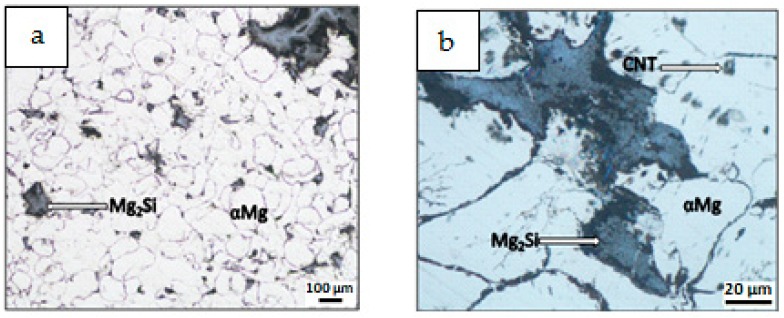
OM micrographs of Mg-Mg_2_Si-CNT composite fabricated from (Mg + nSi_D_) + CNT_D_ powder mixture: (**a**) Mg_2_Si agglomerates in Mg matrix, (**b**) phases of different size and morphology.

**Figure 12:**
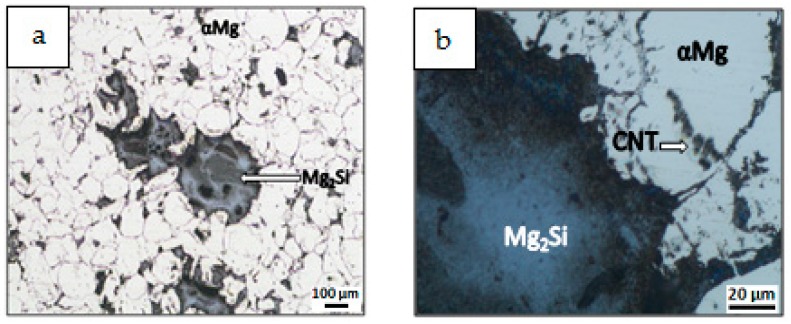
OM micrographs of Mg-Mg_2_Si-CNT composite fabricated from (Mg + CNT_D_) + nSi_D_ powder mixture: (**a**) Mg_2_Si agglomerates in Mg matrix, (**b**) phases of different size and morphology.

Results of composite microstructure examinations with the OM without additional etching are shown in Figure 9, Figure 10, Figure 11 and Figure 12. Two main structural elements were revealed: rounded magnesium areas (light) with characteristic MgO boundaries derived from the initial magnesium powder, and an irregular dark blue phase of similar or greater size (Figure 9a, Figure 10a, Figure 11a and Figure 12a). At higher magnification (Figure 9b, Figure 10b, Figure 11b and Figure 12b), it is visible that the irregular dark blue phase contains very fine black and elongated phases, and this suggests that the particles are a mixture of Mg_2_Si and CNTs. Similar black elongated phases were detected in the initial magnesium powder grains except the oxides (beige), and inside the Mg grains as well. Within the Mg grains, light blue, irregular and very fine precipitations of Mg_2_Si can be observed, and that suggests the SHS reaction. Moreover, that explains the movement of CNTs, previously deposited on the microsized Mg powder, in the magnesium matrix. The comparison of the microstructure of all composites indicates that in the material obtained from the Mg + (nSi_D_ + CNT_D_) mixture, the Mg_2_Si phase is the finest (Figure 9), and more beneficial conditions for matrix reinforcement formation are created.

**Figure 13:**
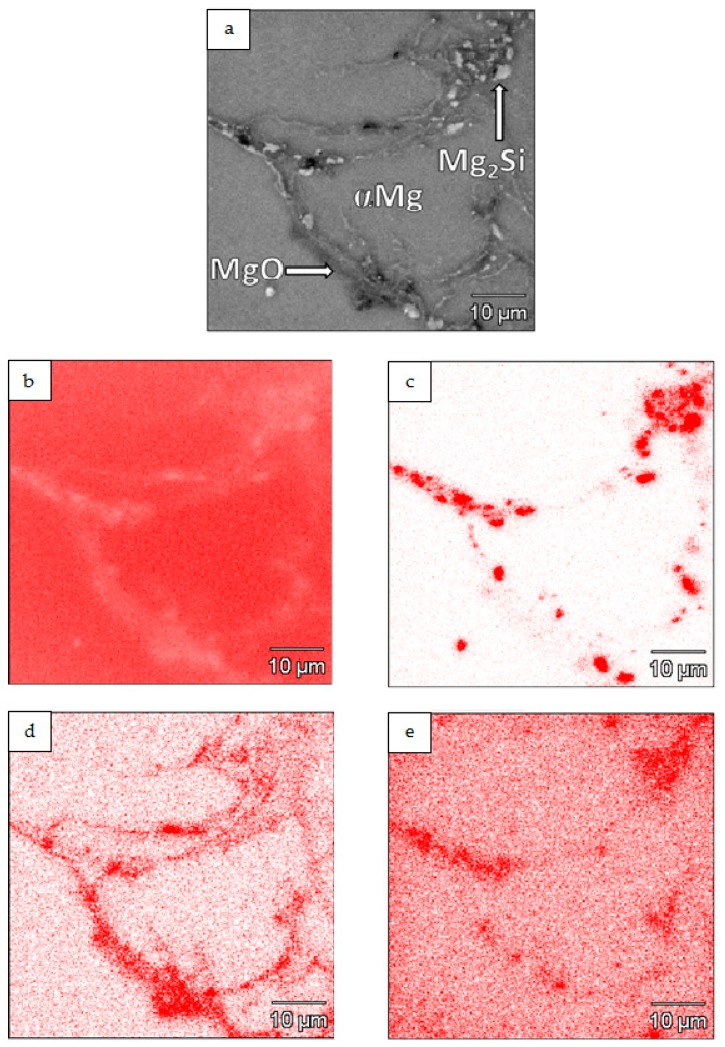
SEM micrographs of Mg-Mg_2_Si-CNT composite fabricated from Mg+(nSi_D_+CNT_D_) powder in composition mode (**a**) with WDS elemental mapping of Mg (**b**), Si (**c**), O (**d**) and C (**e**).

Results of composite microstructure examinations with the SEM and WDS are presented in Figure 13. The results revealed distribution of Mg, Si, O and C. The overlapping areas, enriched in Mg and Si, confirmed in situ formation of Mg_2_Si particles in the composite. Moreover, in the regions containing Mg_2_Si particles, an increase of carbon concentration was observed on the carbon elemental mapping (Figure 13e), which may suggest the presence of CNT’s. Furthermore, the microareas with higher concentration of oxygen were found in the magnesium matrix. The oxygen distribution indicates oxides presence originating from the initial Mg powder surface. Obtained WDS elemental mapping results are consistent with the OM and XRD results.

Results for the porosity and microhardness measurements are presented in Table 2, and the differences depending on the mixing procedure and the effectiveness of the de-agglomeration of the nanocomponents are demonstrated. The lower porosity of the composite obtained from the initial powder mixture with the uniform nanocomponents distribution, that is, Mg + (nSi_D_ + CNT_D_), indicated that this type of powder mixture morphology is the most effective in material compaction, and it influences the composite microhardness, which is a little bit higher for a sinter obtained from this mixture. The highest porosity was obtained for the reference Mg sinter, in comparison with the composites obtained. This result can be explained by the impact of an exothermal reaction between Mg and nSi, which induced a local increase in the temperature in the composite sinter, and better compaction under the same pressure applied.

**Figure 14:**
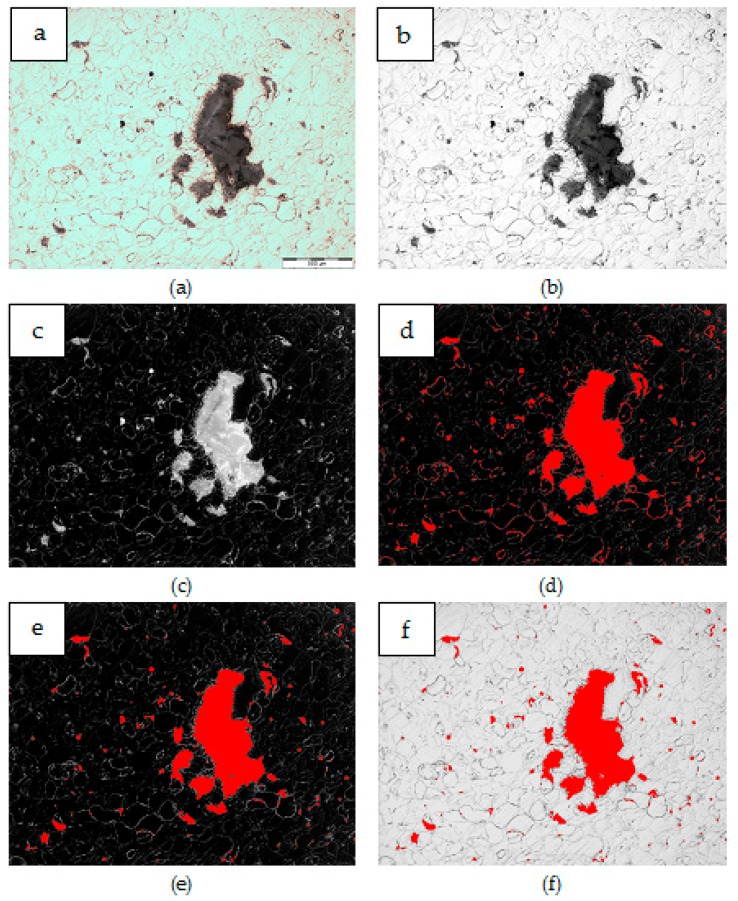
Procedure applied for Mg_2_Si particles detection in Mg-Mg_2_Si-CNT composite sinters: (**a**) initial OM image, (**b**) grey image-histogram equalization, (**c**) numerical inversion, (**d**) automatic binarization (k-means; white phase), (**e**) opening 2 and (**f**) complete image - initial + binary.

**Figure 15:**
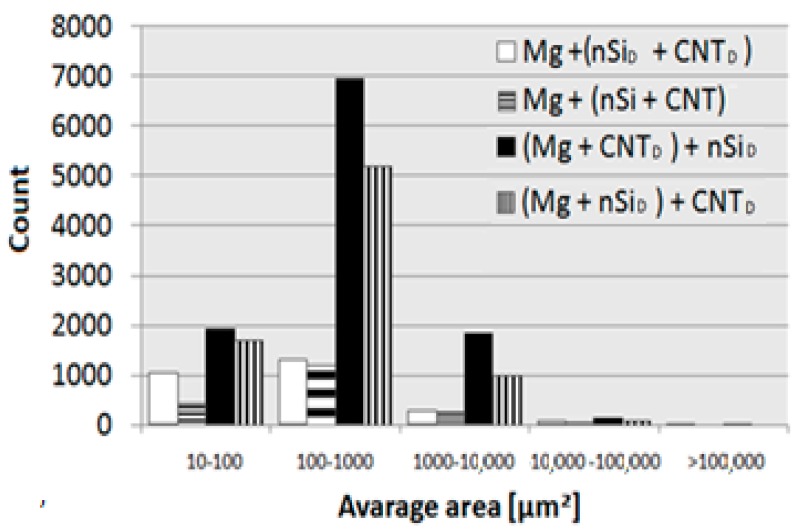
Quantity of Mg_2_Si particles in composite sinters divided into size classes.

An example of the image transformation procedure applied for the quantitative metallography examination of the Mg_2_Si phase with Met-Ilo software is presented in Figure 14. For each sample, 15 areas were measured, and the results are presented in Table 3 and in Figure 15.

Differences in Mg_2_Si synthesis depending on the preparation of the powder mixture were revealed. The main difference was in the value of the silicide area fraction A_A_, which is two times lower for the composites obtained from Mg + (nSi + CNT)_D_ and Mg + (nSi_D_ + CNT_D_) mixtures in comparison to (Mg + nSi_D_) + CNT_D_ and (Mg + CNT_D_) + nSi_D_. That effect was obtained for the same initial powder composition and sintering parameters, which suggests that very fine Mg_2_Si particles smaller than 10 µm^2^ were formed, and many below the OM resolution. The analysis of the detected number of particles in size classes also exhibited an evident difference for the composites from the (Mg + nSi_D_) + CNT_D_ and (Mg + CNT_D_) + nSi_D_ mixtures. For those composites, the number of particles was greater compared to the two other samples. This effect was directly connected to the morphology of the mixture of the initial powders, and it can be explained as a result of the Mg reaction with few micrometer-sized nSi/CNT agglomerates, located either on the Mg microsized powder surface or separately from the metallic particles (Figure 6 and Figure 7).

The preliminary research on the hybrid composite obtained with powder metallurgy showed that the parameters applied for Mg-nSi-CNT sintering and the mixture composition required optimization.

From the literature [13,14,21,22,23] it is known, that due to CNT’s tendency for agglomeration, an application of more than 1 vol.% of CNT’s may cause a decrease of mechanical and electrical properties in metal matrix composite. Thus, in the present work, the highest effective reinforcing amount of fibrous carbon nanocomponent was used. In our previous experiments, the procedure of Mg-nSi-CNT mixture preparing with proposed method was successfully examined to volume fraction less than 2% of nSi and 1% of CNT. However, the effects related with Mg_2_Si formation can be useful in CNT de-agglomeration processes. Therefore, in further experiments two main aspects should be considered. The first aspect is the ratio of nanosilicon to CNT in the powder mixture, and the maximum number of nanocomponents, which can introduced into the Mg-base powder mixture. The second important issue is the adjustment of sintering parameters such as heating speed, sintering temperature and pressure.

Generally, the experiment revealed the possibilities of a design for micro- and nanopowder mixture morphology, and its influence on the final product microstructure and properties, including ex situ and in situ nano-reinforcement in composites.

## 4. Conclusions

A novel approach for processing ternary powder mixtures intended for fabricating hybrid composites, which consisted of a microsized component and two nanosized components, was presented in this work. The main conclusions are drawn as follows:

1. The fabrication of the microsized powder, nanopowder and nanotube mixture was successfully tested for the Mg-nSi-CNT system, and the ultrasonic method for de-agglomerating the nanocomponents in liquid base suspension proved to be useful if the appropriate order of technological procedures has been preserved.
2. The final ternary powder mixture consisted of a microsized powder coated with two nanocomponents, and the components were uniformly distributed when the nanocomponents were first de-agglomerated separately, and then mixed together and deposited at the microsized powder surface.
3. Differences in the microstructure of the Mg-Mg_2_Si-CNT composite depending on the initial morphology of the powder mixture were observed. The most noticeable changes were in the size of the Mg_2_Si particles, where the values could be very high (a few dozen micrometers in diameter), when nSi re-agglomeration occurred. Simultaneously, the size of the CNT agglomerates, detected in the composite, often surrounded by the Mg_2_Si phase, was also greater.
4. The procedure for preparing the Mg-nSi-CNT mixture, which was proposed as the most effective, ensured the lowest porosity and the highest hardness of the Mg-Mg_2_Si-CNT composite obtained by sintering under pressure.

## Figures and Tables

**Table 1:** Applied procedure of ternary powder mixture preparation and condition of their sintering process (subscript D means the ultrasonic de-agglomeration in alcohol).

Mixture Signature	Mixture Composition [vol.%]	Mixing Procedure	Sintering Conditions
Mg	100 Mg	Reference sample	580 °C, 15 MPa, vacuum
Mg+(nSi_D_ + CNT_D_)	97% Mg; 2% nSi; 1% CNT	- separate de-agglomeration nSi and CNT - preparation of (nSi)_D_ + (CNT)_D_ suspension - addition of Mg to (nSi)_D_ + (CNT)_D_ suspension	
Mg + (nSi + CNT)_D_		- common de-agglomeration of nSi and CNT - addition of Mg+(nSi+CNT)_D_ suspension	
(Mg + nSi_D_) + CNT_D_		- separate de-agglomeration of nSi and CNT - preparation of (nSi)_D_ + Mg suspension - addition of (nSi)_D_ to Mg + (CNT)_D_ suspension	
(Mg + CNT_D_) + nSi_D_		- separate de-agglomeration of nSi and CNT - preparation of (CNT)_D_ + Mg suspension - addition of (CNT)_D_ to Mg + (nSi)_D_ suspension	

**Table 2:** Hardness and open porosity of composite sinters obtained from Mg-nSi-CNT mixtures of the same composition and prepared by different way.

Material	Hardness, HV 0.2	Open Porosity, %
Mg	41.98 ± 2.8	1.22
Mg + (nSi_D_ + CNT_D_)	46.5 ± 3.0	0.71
Mg + (nSi + CNT)_D_	44.5 ± 2.7	1.05
(Mg + nSi_D_) + CNT_D_	43.6 ± 4.7	0.88
(Mg + CNT_D_) + nSi_D_	44.6 ± 3.9	0.76

**Table 3:** Quantitative characteristics of Mg_2_Si particles sized of more than 10 µm^2^ formed in Mg-Mg_2_Si-CNT composite sinters.

Material	Area fraction, A_A_ [%]	Area [µm^2^]	Count	Count
Mg + (nSi_D_ + CNT_D_)	5.38 ± 2.66	10–100	747	2367
		100–1000	1295	
		1000–10,000	272	
		10,000–100,000	49	
		> 100,000	4	
Mg + (nSi + CNT)_D_	6.69 ± 4.11	10–100	441	1950
		100–1000	1198	
		1000–10,000	246	
		10,000–10,0000	52	
		> 10,0000	13	
(Mg + nSi_D_) + CNT_D_	18.75 ± 9.88	10–100	1708	8018
		100–1000	5208	
		1000–10,000	994	
		10,000–10,0000	97	
		> 100,000	11	
(Mg + CNT_D_) + nSi_D_	17.50 ± 2.59	10–100	1917	10814
		100–1000	6935	
		1000–10,000	1833	
		10,000–100,000	122	
		> 100,000	7	
